# Properties of Cementitious Materials Utilizing Seashells as Aggregate or Cement: Prospects and Challenges

**DOI:** 10.3390/ma17051222

**Published:** 2024-03-06

**Authors:** Yunpeng Zhu, Da Chen, Xiaotong Yu, Ruiwen Liu, Yingdi Liao

**Affiliations:** 1 College of Harbor, Coastal and Offshore Engineering, Hohai University, Nanjing 210098, China; zhu0818@hhu.edu.cn (Y.Z.); chenda@hhu.edu.cn (D.C.); xiaotongyu@hhu.edu.cn (X.Y.); liuruiwen@hhu.edu.cn (R.L.); 2 Key Laboratory of Coastal Disaster and Defense of Ministry of Education, Hohai University, Nanjing 210098, China; 3 Yangtze Institute for Conservation and Development, Hohai University, Nanjing 210098, China

**Keywords:** seashell, concrete, durability, mechanical properties, physical properties

## Abstract

Nowadays, the sustainable development of the construction industry has become a focus of attention. Crushing and grinding waste seashells originating from the fishery industry, such as oyster shells, cockle shells, mussel shells, and scallop shells, into different particle sizes for usage as aggregate and cement in concrete or mortar provides an effective and sustainable solution to environmental problems by reducing natural resource dependence. Numerous studies have attempted to analyze the suitability of waste seashell as a possible alternative to natural aggregates and cement in concrete or mortar. This paper presents an up-to-date review of the characteristics of different types of waste seashell, as well as the physical, mechanical, durability, and other notable functional properties of seashell concrete or mortar. From the outcome of the research, waste seashell could be an inert material, and it is important to conduct a series of proper treatment for a better-quality material. It is also seen from the results that although the mechanical properties of seashell concrete have been reduced, they all meet the required criteria set by various international standards and codes. Therefore, it is recommended that the replacement of seashells as aggregate and cement should not exceed 20% and 5%, respectively. Seashell concrete or mortar would then have sufficient workability and strength for non-structural purposes. However, there is still a lack of investigation concerning the different properties of reinforced concrete members using seashells as the replacement of aggregate or cement. Further innovative research can solidify its utilization towards sustainable development.

## 1. Introduction

Concrete is one of the most versatile building materials in the world. Due to its low price, easier maintenance, long service life, high strength, and ability to withstand harsh weather conditions, concrete is rapidly increasing in use every year and is the second most consumed material in the world [1]. Research shows that the annual consumption of concrete in industrial society exceeds 10 billion tons [2,3]. As the world’s population increases, the use of concrete will increase to about 18 billion tons per year [2,4]. Concrete is a composite material consisting mainly of water, cement, and aggregates. According to statistics, the concrete industry needs 1.5 billion tons of cement and nearly 20 billion tons of aggregates for concrete production every year [5,6]. With the increase in demand, the extraction of natural raw materials such as river sand has risen sharply. This imbalance of supply and demand has caused great harm to the environment. Authorities in some parts of the world have imposed restrictions on the mining of natural materials through taxation [7,8]. In order to develop in a sustainable direction, a lot of research has been carried out to reduce the problem of excessive consumption of traditional materials by replacing traditional materials in concrete with waste materials originating from different sources, such as waste shells [1,9], glass [10], rubber [11], and coral reefs [12].

Like Japan, South Korea, and many Southeast Asian coastal countries, China has very rich marine resources. Research statistics show that China is the leading producer of shellfish, with an output of 15 million tons in 2020. Japan is the second largest producer, followed by the US, South Korea, Thailand, France, and Spain. However, with little commercial value, only a small portion of this enormous waste is recycled to reuse [13]. Waste is mostly dumped randomly in open fields. Prolonged storage of untreated shellfish waste can cause microorganisms to break down the salt into gases such as hydrogen sulfide, ammonia, and amines [7]. This can produce unpleasant odors and flies, ultimately leading to serious environmental and public health problems [14]. If these wastes cannot be effectively removed, the adverse impact on the environment will become increasingly irreversible.

**Figure 1:**
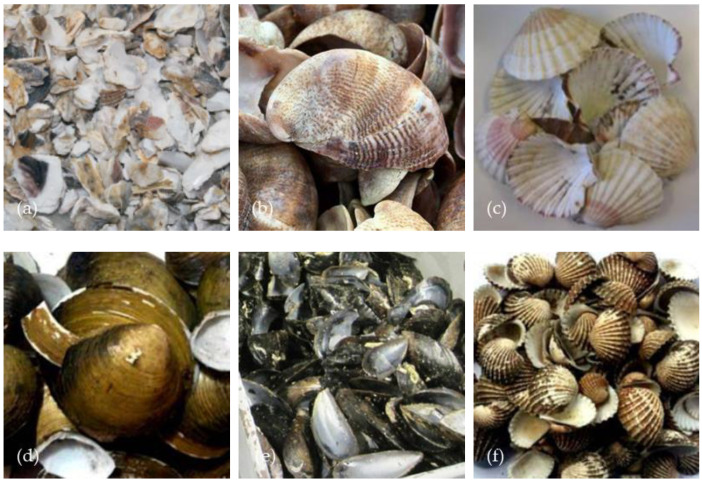
Different types of seashells: (**a**) oyster, (**b**) crepidula, (**c**) scallops, (**d**) clam, (**e**) mussel, (**f**) cockle [20,21,22,23].

Over the past three decades, researchers have proposed many solutions to this problem. For example, waste oyster shells have been used in agricultural fertilizer preparation, water treatment agents, and soil adsorbents. However, their high production cost and low availability limited the widespread use of these methods [15,16,17,18]. In addition, high energy consumption and pollution further limited the use of seashells in soil sorbent production [13,19]. Therefore, it is crucial to develop cost-effective, environmentally friendly, and sustainable approaches to tackle the growing seashell problem. In recent years, chemical analyses have shown that more than 90% of calcium carbonate is found in seashell components, and in cement-based materials, calcium carbonate can be classified as an inert material [7]. Therefore, they can be recycled and processed into fine aggregates, coarse aggregates, or cement substitutes. Figure 1 shows the different types of seashells used to replace aggregates or cement in this paper. Firstly, it is an efficient and more economical way to dispose of this waste. In addition, the use of seashells as an aggregate or cement substitute in concrete will reduce reliance on natural raw materials. Therefore, adding seashells to concrete can protect the natural resources that produce concrete and reduce environmental problems caused by over-exploitation.

Over the past two decades, especially in the last five years, a great deal of research has been carried out. Firstly, the properties of different kinds of seashells were studied, including physical properties [20,23,24,25], chemical components [20,25,26,27], and microstructure [20,28,29,30,31]. Secondly, the cleaned, crushed, dried, and calcined seashell particles or powders were used as aggregates [32,33,34,35] or cement [36,37,38,39] substitutes and added to concrete and mortar. Finally, scholars investigated the physical [28,29,40,41], mechanical [33,40,42,43,44], and durability [35,38,45,46,47] properties of seashell concrete and mortar by configuring concrete and mortar with different replacement rates to determine the optimal replacement rate and provide guidance for engineering practice.

Therefore, the research work on seashell concrete and mortar needs to be summarized to guide future researchers and potential builders. This paper is based on research carried out over the last two decades and begins with a survey of scholarly research directions and priorities in the field of seashell concrete and mortar through the VOSviewer (version 1.6.18) tool. Secondly, focusing on the latest research results in recent years, this paper comprehensively analyses the feasibility of waste seashells as aggregate and cement in concrete or mortar, respectively, and their effects on physical, mechanical, and durability properties of concrete and mortar. In this way, the optimal level of seashells as aggregate and cement is summarized. At the end of the paper, some directions for future investigations in conjunction with the gaps in previous research are presented. In addition, this paper summarizes the current research on the eco-efficiency and cost efficiency of seashell concrete, which is in line with the current concept of sustainable development in the field of construction around the world.

## 2. Review Methodology

The present review aims to explore the potential of waste seashells in the production of concrete or mortar. To this end, the following research issues have been formulated to find articles with a higher correlation with the present review:

(1) What are the differences in the physical and chemical properties of waste seashells as aggregate and natural aggregate?
(2) Why is there an urgent need to investigate the possibility of using waste seashells in concrete or mortar?
(3) What are the fresh and hardened properties of seashell concrete or mortar?
(4) How does seashell concrete or mortar differ from ordinary concrete in terms of durability?
(5) What is the contribution of the use of waste seashells in concrete to sustainable development?

The Preferred Reporting Items for Systematic Reviews and Meta-Analyses (PRISMA) methodology was used in the present review [48]. The core of this methodology is to find a reliable search engine. It is well known that Google Scholar, Scopus, Web of Science, Science Direct, etc., are very trustworthy databases for paper searches. Therefore, the present review used Web of Science (WoS) for the literature collection and then evaluated and filtered the papers. In the initial phase, the literature was collected by keyword, and as of January 2024, a search of the WoS database for “waste seashells in concrete and mortar” was supplemented by the names of various common seashells. After a series of evaluations and screening, 422 relevant papers were identified and 99 of them were selected as the key references for the present review.

VOSviewer (version 1.6.18) is a software tool for constructing and visualizing bibliometric networks. These networks may, for instance, include journals, researchers, or individual publications, and they can be constructed based on citation, bibliographic coupling, co-citation, or co-authorship relations.

**Figure 2:**
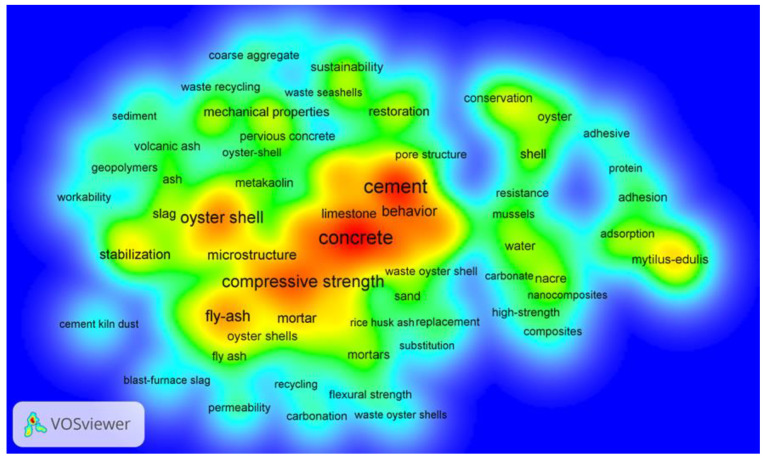
Co-occurrence of keywords (density mapping).

The relevant literature obtained in the WoS database was exported as tab-delimited files and loaded into VOSviewer for keyword analysis. Table 1 lists the 10 most common keywords in the literature on the subject area of seashell concrete. The analysis showed that scholars currently favored the use of waste seashells as a substitute for natural aggregates in concrete or mortar in order to achieve economic and ecological sustainability. There was also a strong interest in the microstructure of the seashells themselves and the mechanical properties of seashell concrete. Figure 2 shows the result of the density visualization for this analysis. Each point on the plot is filled with a color based on the density of the elements surrounding that point, with higher densities being closer to red and, conversely, lower densities being closer to blue. The density size depends on the number of elements in the surrounding area and the importance of those elements. It is easy to observe that compressive strength, oyster shells, concrete, and aggregates are the densest.

The density visualization can be used to quickly view the density of knowledge and research in this domain. Based on the analysis of publications and keywords, the resulting research highlights on seashell concrete are all described in detail in the following sections.

## 3. Properties of Seashells

### 3.1. Physical Properties

Researchers around the world have conducted extensive experiments with waste seashells in the form of seashell aggregates and seashell powder. The physical, mechanical, and durability properties of seashell concrete are largely determined by the properties of the aggregates and powders that make up the shell. The properties of seashells from the available literature have been presented in Table 2.

The specific gravity of any material is the ratio of the density of the particular material to that of water. In general, crushed seashells were used as fine aggregate with sizes of less than 5 mm. On the other hand, when they were used as coarse aggregate, they were processed with a maximum size of between 16 and 25 mm. However, when incorporated in pervious concrete, Martínez-García et al. [20] and Khankhaje et al. [24] claimed that aggregates between 4 and 9.5 mm in size can also be used as coarse aggregate. The specific gravity of coarse and fine seashell aggregate varies in the range of 2.09–2.67 and 2.10–2.73, respectively. Khankhaje et al. [24] displayed the lowest specific gravity value of 2.09 for coarse aggregate, while Kuo et al. [50] showed the same value of specific gravity for fine aggregate. The highest value of specific gravity for coarse aggregate was reported by Eo and Yi [23] to be 2.67, while Martínez-García et al. [20] displayed the highest specific gravity of 2.73 for fine aggregate. The specific gravity of seashell aggregates is usually lower than that of natural aggregates. The researchers found through testing that the specific gravity of natural coarse and fine aggregates varied in the range of 2.51–2.87 and 2.58–2.83, respectively. Although some of the seashells were outside the ACI limits for normal weight aggregates used in concrete (2.30–2.90), such as some oyster shells and cockle shells, the specific gravity of all seashells was above the ACI recommendations for light aggregates.

A significant variation in the water absorption of seashell aggregates was observed, depending on the presence of an irregular surface and number of internal pores [28], as seen in Table 2 Under normal circumstances, the water absorption of normal aggregates is less than 2% [52], and the maximum water absorption recommended in ACI cannot exceed 8%. Studies showed that the water absorption of coarse aggregate is lower than that of fine aggregate. They did not vary much, usually between 1.88 and 8.87%. But in some studies, the authors gave different results. Eo and Yi [23] found that oyster shell aggregates up to 25 mm had a water absorption of 0.4% and Falade [53] (not listed in Table 2) found that the water absorption of periwinkle shell aggregate was up to 12.99%. The water absorption of aggregates has an influence on the workability and consistency of concrete or mortar. Therefore, it is necessary to specify the amount of water absorption of seashell aggregates required for effective mix design.

In some past studies, waste seashells were also ground into powder to be a replacement for cement. The results showed that the specific gravity of seashell powder was generally lower than that of OPC (3.10), and the particle size depended on the temperature of the calcination and grinding processes. Lertwattanaruk et al. [25] found the specific gravity of clam shell, mussel shell, oyster shell, and cockle shell powder to be 2.71, 2.86, 2.65, and 2.82, respectively. By grinding oyster shells in wet and dry methods, Zhong et al. [54] obtained different median sizes with D50 of 1.61 and 58.53 μm, respectively. Ez-Zaki et al. [55] achieved powder of 6.27 and 10.22 μm by milling the same seashell type. From the study by Lertwattanaruk et al. [25], it can be found that the average particle sizes of Portland cement and the clam, mussel, oyster, and cockle shells were 22.82, 20.80, 29.87, 13.93, and 13.56 μm, respectively, which corresponded to a specific surface area of 3376, 8279, 6186, 14,280, and 8299 cm^2^/g, respectively. Compared to Portland cement, seashell powder has a greater specific surface area after processing, making it more reactive for the cementitious material to react with other substances to form a binder with appreciable strength.

### 3.2. Chemical Composition

The chemical composition of seashells varies depending on the type of shells and where they were collected. Most researchers calcined shells to study their chemical composition. Table 3 lists the chemical composition of the raw shells and the shells after calcination. It is obvious that there is no significant difference in the original chemical composition of oyster shells collected from rivers and the sea, except that the river oyster is slightly higher in calcium carbonate content. The data measured by Abinaya and Venkatesh [26] confirmed this regularity. There is no obvious difference in the chemical composition of different types of shells, all of which are composed of calcium carbonate and a small number of other oxides, and the calcium carbonate content of most shells is more than 95%. Apparently, the shells after calcination contained higher calcium oxide, which suggests that seashells could be an inert material in concrete and mortar, similar to limestone.

It is reported that in order to reduce impurities, organic matter, and salt content, especially chloride ions, seashells need to be washed before reusing [20]. Chloride ions and sulfates in seashells prevent the effective bonding of aggregates to cement matrix, thereby affecting the setting properties and ultimate strength of concrete. The percentages of organics and chloride ions in untreated seashell aggregates often exceed the maximum values allowed for conventional concrete [20,22,58]. The excessive chloride content in concrete could accelerate the corrosion of steel reinforcement, while excessive sulfate content could trigger the expansion of hardened concrete.

Differences in calcium oxide content in shells after calcination depend mainly on the type of shells, cleaning method, and the method or temperature of the calcining treatment. Felipe-Sese et al. [27] calcined at 1100 °C to obtain shells with a calcium oxide content as high as 87.21%. For the same type of seashell (mussel shell), Lertwattanaruk et al. [25] obtained only 53.58% calcium oxide in shells at a calcination temperature of 550 °C. Therefore, in general, all types of seashells have similar chemical compositions when similar calcination temperatures are employed.

### 3.3. Microstructure

**Figure 3:**
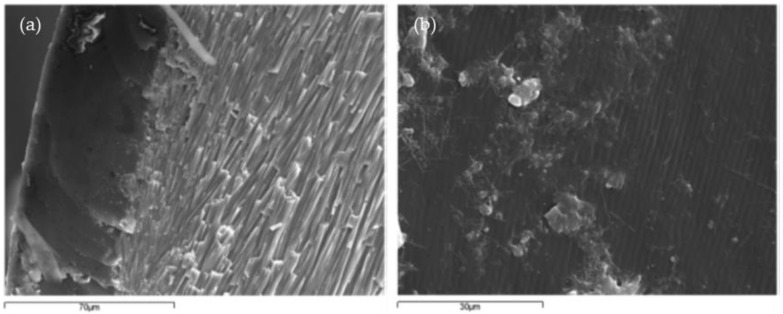

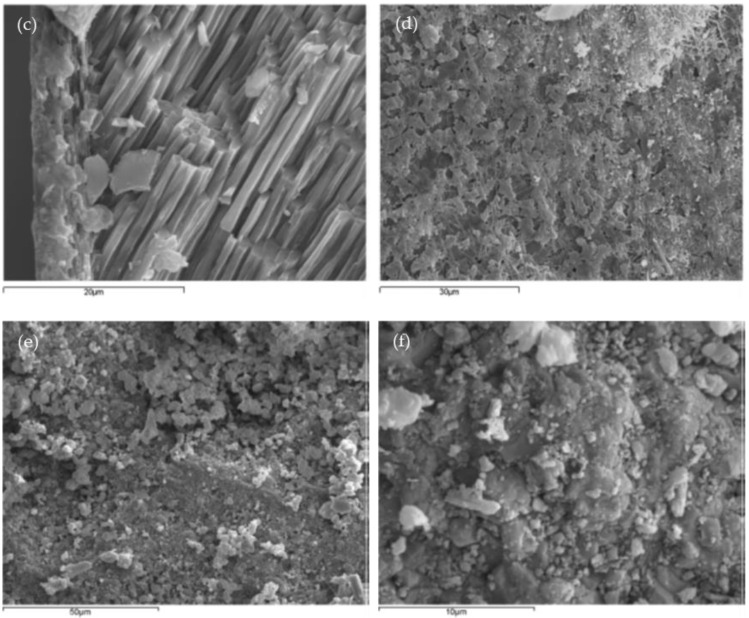
SEM analysis of mussel shell composition by Martínez-García et al. [29]: (**a**) periostracum (external layer)—prismatic structure layer; (**b**) periostracum layer front view; (**c**) prismatic structure layer; (**d**) nacre layer front view; (**e**) nacre layer; (**f**) limestone particle.

According to the study by Martinez-Garcia et al. [20], the structure of mussel shells can be divided into three parts: the outer layer called periostracum, the middle layer called the prismatic layer, and the inner layer referred to as nacre [20,64,65]. Most of the other species of seashells were also made up of these three parts. The periostracum is unmineralized and consists mainly of proteins. Its morphological characteristics are shown in Figure 3a,b. The central and thicker layer (approximately 400 mm) has an array of parallel prisms with polygonal cross-sections and its main component is calcium carbonate, as shown in Figure 3c. The last layer, about 10 mm wide, consists of layers of aragonite parallel to the surface, as shown in Figure 3d,e.

**Figure 4:**
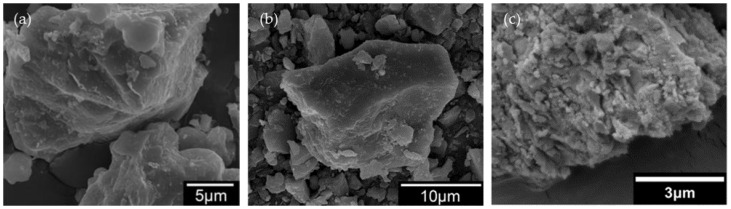
Particle surface morphology of (**a**) limestone powder; (**b**) Portland cement powder; (**c**) seashell powder [28].

When seashells were ground into powder for cement replacement, Wang et al. [28] observed the surface morphology of seashell powder, limestone powder, and cement powder particles by SEM. From Figure 4a,b, it can be seen that the surface textures of limestone powder and cement powder are relatively smooth, while the surface of seashell powder particles has many tiny protrusions, irregularities, and walls. This explains why seashell powder has a larger surface area compared to limestone powder and cement powder. This positively affects the rheological properties, hydration development, and mechanical strength.

**Figure 5:**
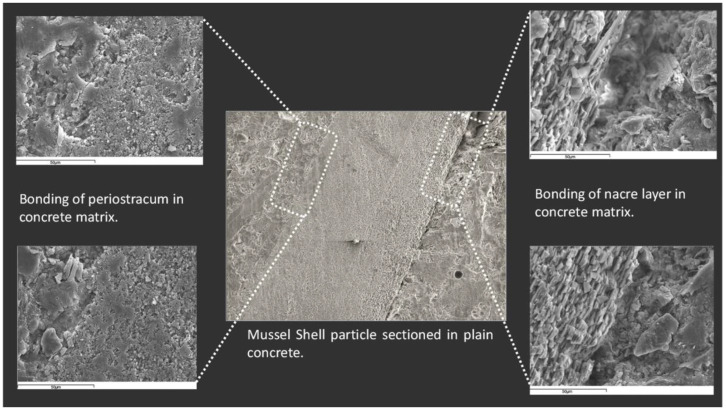
SEM observation of microstructure of seashell concrete [20].

**Figure 6:**
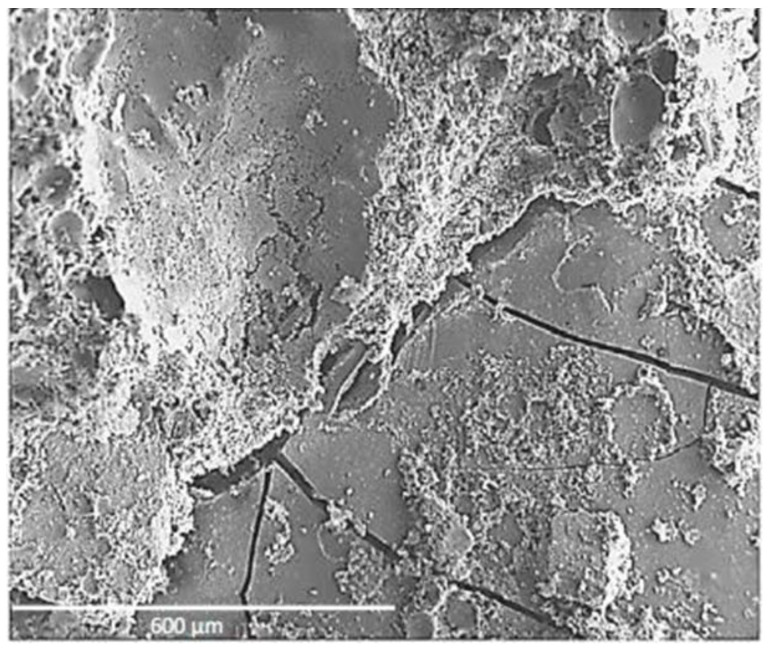
Cracks in seashell particles and cement paste [30].

Martinez Garcia et al. [20] found that when seashells were added to concrete in the form of aggregates, it reduced the bonding of seashell aggregates, which is especially enhanced with coarse aggregates. Cracks and pores were found in the interfacial transition zone with the periostracum in the scanning electron microscope (SEM) image (Figure 5), while the interfacial transition zone (ITZ) with the nacre layer showed a complete lack of bonding and very high porosity. Similarly, some researchers [30,50] also observed through SEM images that the use of seashells as aggregate resulted in poor cement paste-aggregate bonding and the creation of a large number of pores. In addition, Martínez-García et al. [30] observed a large number of cracks in the mortar at 28 days (Figure 6). The researchers found that the use of seashells as a mixing material in concrete did not produce unusual chemical reactions or new substances [23,49].

**Figure 7:**
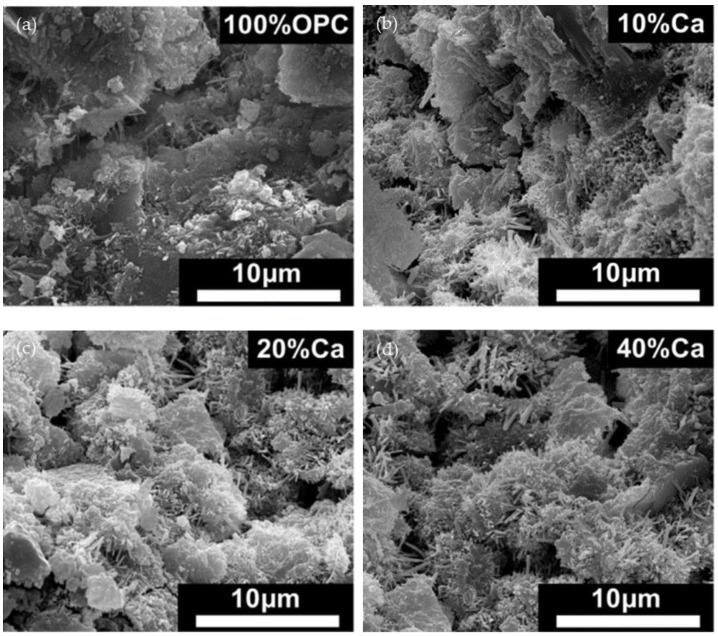
SEM of hydrated cement matrix produced in seashell cement mixtures [28]: (**a**) 100% OPC; (**b**) 10%Ca mixture; (**c**) 20%Ca mixture; (**d**) 40%Ca mixture.

As can be seen in Figure 7a, the most common hydration products in 100% ordinary Portland cement typically consist of C-S-H, Ca(OH)_2_ and ettringite. However, in blended cement mixtures containing seashell powder, ettringite-, and calcium carboaluminate-like phases appear near the seashell powder. And it increases with the increase in amount of shellac in the mixture [28].

## 4. Preparation of Seashell Mortar or Concrete

### 4.1. Treatment of Waste Seashell

In past studies, waste seashells needed to be washed and dried before they could be reused. Some researchers also calcined them at high temperatures [32,33,36,42,47,60,66,67,68]. They were then crushed or ground into granules or powder as required. Among them, the cleaning requires the removal of the organic matter and salt in the seashells to meet the requirements of traditional concrete for sulfate content. Drying or calcining are used to dehydrate and sterilize the seashells, further reducing the organic content. The equipment used by the researchers to grind seashells varied; the most common was a drum compactor [41], jaw crusher [25,69,70], and hammer [58]. The seashells used as fine aggregate, coarse aggregate, and cement in concrete also varied in size. Generally, the seashells used as fine aggregate were crushed and sieved below 5 mm. The seashells used to replace coarse aggregates were usually either uncrushed [71] or crushed to around 10 mm [20,24]. The seashells used as cement substitution were usually crushed and ground to below 30 µm.

Typically, the seashells were washed in cold water, and Chakravarthy and Mutusva [72] also performed a secondary cleaning in hot water containing vinegar. In addition to cleaning, Soneye et al. [73] used domestic brushes to manually remove impurities and other organics. Usually washed seashells are dried at 100–110 °C for 24 h [32,33,47]. After washing and drying, some researchers also calcined the seashells. According to Ibrahim et al. [36], after calcination at 500 °C for 24 h, atomic absorption column analysis showed that the calcium ratio increased to 58%. The calcium content reached 68% after 48 h of the continuous burning process, and when the calcination process continued for 96 h, the calcium percentage rose to 90%. The relationship between the pH value of seashell and calcination temperature was derived by Chiou et al. [37]. It can be found that the pH value of seashell and chloride content improved with increasing calcination temperature and that the improvement was not significant when the temperature exceeded 650 °C. Furthermore, calcination [37] found that the CaO contents were 64.89%, 66.81%, 73.45%, 74.96%, and 78.56% at calcination temperatures of 550 °C, 650 °C, 750 °C, 950 °C, and 1050 °C, respectively. Similarly, when the calcination temperature continued to increase after a certain point, the increase in the CaO content of the seashells was not very significant. Therefore, from a sustainable point of view, increasing the temperature and prolonging the calcination time cannot bring about further improvement in the properties of the shells. Increasing the calcination temperature could accelerate the generation of hydration products, fill the pore structure of concrete or mortar, and reduce its rate of water absorption [38]. In addition, other alternative calcination temperatures and durations for shells proposed by other researchers were 800 °C for 1 h [60], 500–800 °C for 3 days [66], 500–600 °C for 3 days [67], and 1000 °C for 1 h [68].

### 4.2. Preparation of Specimens

In past studies, the design, preparation, and casting of concrete mix containing waste seashells were similar to those of ordinary concrete and were carried out according to various standard specifications. When preparing mortar or concrete specimens, batching was carried out by weight, and only a few studies determined the water–cement ratio by volume. Based on a review of the previous literature, taking into account the different replacement rate of seashell and their different water absorption compared with natural aggregate, the water–cement ratio ranged from 0.3 [42] to 0.8 [53,74] when preparing the specimens. Different water–cement ratios would affect the durability properties such as drying shrinkage and porosity of seashell concrete and mortar. Since the water absorption of shells as aggregates or cement is higher than that of natural materials, many researchers added additional water during batching [20,41]. The percentage substitution levels by mass of traditional fine or coarse aggregate with seashell were between 5–75%, and the replacement rate by mass for cement was between 0–50%. The different replacement rates mainly affected the mechanical properties of seashell concrete and mortar. Table 4 gives the details of the preparation of specimens in some literature. If the specimen is mortar, the mix proportions in the table represent cement, namely fine aggregate, and if the specimen is concrete, it is cement: fine aggregate, namely coarse aggregate. After casting, it was removed from the molds after curing for 24 h. The specimens were then cured in water (temperature range from 20 to 26 °C [36]) until the corresponding test age was reached.

### 4.3. Other Component Materials

In the reviewed papers, ordinary Portland cement (OPC) according to ASTM [79] C150-07 type I was the most commonly used binder. The strength grades of these cements were usually 42.5R [32,40,47] and 52.5R [80]. Most studies did not add any other materials in OPC. Nevertheless, Liao et al. [40] replaced about 11% of the weight of cement with metakaolin (MK). When preparing crushed waste oyster shell mortar, Chen et al. [32] used fly ash (FA) and ground granulated blast furnace slag (GGBS) as supplementary cementitious materials (SCMs). Ahsan et al. [77] added silica fume as an additive to cement. The use of these additives would evidently increase strength and durability, thus decreasing permeability and shrinkage caused by particle packing [81]. These additives also promoted hydration and resulted in a denser microstructure [82].

In these studies, the normal fine aggregates included river sand [32,33], Pumice-type natural (volcanic) pozzolan [63], alluvial quartz sand [23], and alluvial silica sand [57]. The natural coarse aggregates included granitic gravel [77], and crushed quartzite [41]. Some researchers treated the aggregates before preparing the specimens. Liu et al. [47] modified crushed aggregates by polyvinyl alcohol (PVA) and sodium silicate (SS), which could improve the durability properties of mortar. Some studies also added other chemical additives such as naphthalene air-entraining water-reducing admixture [49], naphthalene sulphonate condensate superplasticizer [83], sulfonated naphthalene formaldehyde (SNF), and superplasticizer [40,47].

## 5. Physical Properties of Concrete and Mortar Containing Seashells

### 5.1. Workability

Workability is a fundamental property of fresh mortar and concrete, which determines its ease of mixing, transport, pouring, and finishing without segregation. The slump test is often used to estimate the workability of mortar and concrete. In most studies, the incorporation of waste seashell as aggregate or cement reduces the slump value of mortar and concrete due to the high porosity and water absorption, angular shape, and rough surface of seashells. Table 5 illustrates the effect of using seashells as a substitute for aggregate or cement in concrete and mortar on their workability.

Edalat-Behbahani et al. [75] reported that the presence of fine particles in sand causes a decrease in the workability of concrete due to their high-water absorption, and these fine particles may coat the aggregates and impair the aggregate–cement paste bond. Ruslan et al. [85] found that substitution of crushed cockle shell content as partial fine aggregate influenced the concrete workability, and the increasing of crushed cockle shell proportion resulted in a drop in slump value. Yang et al. [49] suggested the use of admixtures to improve workability. After adding a naphthalene air-entraining water-reducing agent, the slump of seashell aggregate concrete increased, but with the increase in replacement rate, the effect of the admixture on the slump decreased. Whereas Adewuyi et al. [84] noted that when up to 75% of the coarse aggregate was replaced with periwinkle shells, the slump was reduced by up to 67%.

Martínez-García et al. [20,30] used spread diameter and penetration depth to represent the workability of mortar and showed that mussel particle shape (with a high percentage of flaky particles) significantly increased water demand, thereby increasing mortar consistency and reducing mortar workability. These results were consistent with those obtained by other authors [25,49,86], which, in all cases, concluded that mixtures made with seashells as aggregates reduced mortar and concrete workability.

Etuk et al. [60] reported that the consistency of the blended cement paste increased as the percentage of cement replaced by seashells increased. In general, the workability of concrete decreases as the percentage of seashell powder increases. On the one hand, the irregular shape of the seashell particles increases the surface area. Therefore, more paste is required to overcome the friction between particles. On the other hand, additional water is required to achieve the desired consistency or inter-particle mobility due to the proliferation of CaO.

According to the study by Liao et al. [87], the initial slump flow of the mortar was found to increase with the increase in the particle size of aggregate, and during a 2 h testing time, the mortar with oyster seashells of large particle size had the smaller slump flow loss. Chen et al. [33] also observed this phenomenon. A possible reason was the larger antiparticle friction resulting from the more irregular surface and larger specific surface area of the particle [88].

Conversely, some researchers gave the opposite result. Workability slightly increased for some mixtures when fine aggregate was partially replaced with crushed shells in concrete at low substitution levels (5–25%) [20,49,57]. Hasan et al. [67] found that the slump flow of concrete increased when 30% of the OPC was replaced by seashell powder, which was because the presence of CaCO_3_ in seashell powder was more than OPC, reducing the density of the concrete that increased the amount of concrete paste. It is therefore recommended that the replacement level of seashells as aggregates in concrete or mortar does not exceed 20% and as cement does not exceed 5% in order to ensure adequate workability.

### 5.2. Setting Time

The setting time is an important reference for assessing the early strength development of concrete or mortar. In most of the studies, the addition of seashells was able to retard the setting time of the concrete or mortar, as shown in Table 6.

According to Liao et al. [87], when the oyster shell fine aggregate was increased up to a replacement level of 20%, the initial setting and final setting time of the mortar would be prolonged, and increased with an increase in particle size. Lu et al. [90] explained that the aggregate of large particle size resulted in the remarkable maintaining of free water available for the hydrolysis, which contributed to an increase in the effective water-to-cement ratio. The cement paste with a higher water-to-cement ratio was known as having better workability and taking a longer time to form a rigid structure. The delay in the setting time of seashell mortar is in agreement with the results obtained in works carried out by other authors [28,30,57].

Soltanzadeh et al. [63] reported that the initial and final setting time increased with the increase in seashell powder content in the cement. As seashell powder increased in the composition of the cement, Portland cement content decreased. Consequently, the surface area of the Portland cement decreased and the hydration process slowed down, which caused a delay in setting time. In other studies, many researchers found that replacing part of the cement with seashell powder prolonged the setting time [25,60,68,75]. However, contrasting findings were reported by Olivia et al. [21], whereby the final setting time was shortened after replacing cement with 4% ground cockle and clam shell powder. Therefore, in order to ensure the strength development of the concrete or mortar in the early stages, it is recommended that the replacement level of seashells as aggregates and cement does not exceed 20% and 5%, respectively. From another point of view, a slow rate of hydration implies the low rate of heat development, which is beneficial for construction (especially rendering and plastering) in hot climates.

### 5.3. Density

The hardened density is one of the most crucial properties of concrete, and the concrete compressive strength largely depends on it. Important characteristics of waste seashells that often affect density are water absorption and specific gravity.

Following the comparative study results of researchers, as the replacement rate of seashell aggregate increased, the density of concrete or mortar slightly decreased, as seen in Table 7. This was mainly due to the irregular shape of the shells and the presence of organic matter which created more entrapped air in the concrete [20,41,55,57]. Chen et al. [33] found that the average hardened density of oyster shell aggregate mortars cured for 90 days was slightly lower than the reference value (0.26–0.69%). The decrease in hardened density can be attributed more to the lower saturated surface dry density of crushed oyster shells (2411 kg/m^3^) than that of river sand (2620 kg/m^3^). Furthermore, the random distribution of crushed oyster shell particles in the mortar also likely led to a looser structure [20]. When fly ash or ground granulated blast-furnace slag was added to the seashell aggregate mortar, the hardened density of the mortar decreased because the specific gravity of FA and GGBS was lower than OPC (3.01) [32]. However, Liu et al. [47] reported an increase in the apparent density of oyster shell mortars treated with sodium silicate, because alkalis usually accelerated the hydration of the cement paste, and the pores in the cement mortar were filled with hydration products. Khankhaje et al. [91] reported that reducing the seashell size can increase the density of the pervious concrete.

### 5.4. Air Content

**Figure 8:**
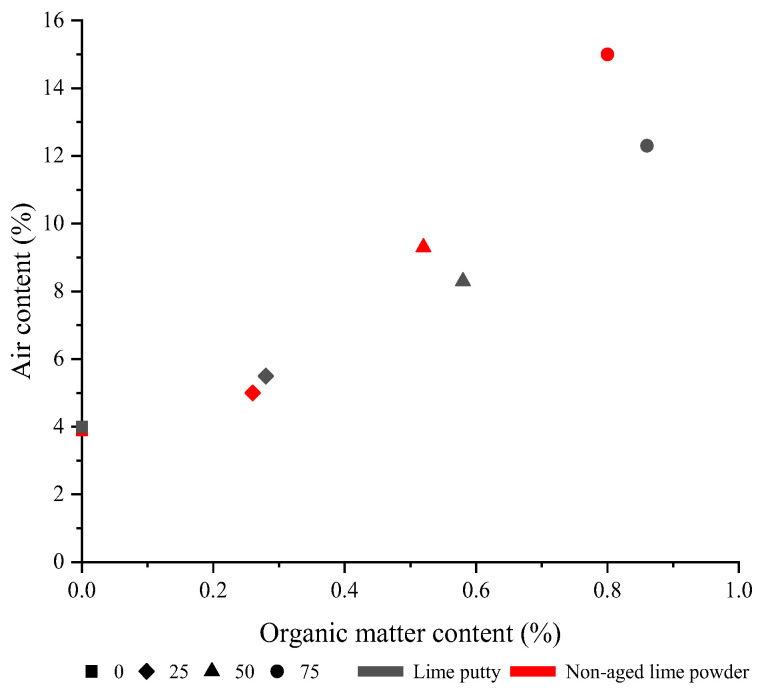
Relationship between air content and organic matter content [29].

Eo and Yi [23] found that when 50% of the aggregate in the concrete was replaced by crushed oyster shells, the air content increased from 2.2% to 5.6%. Martínez-García et al. [30] found that the air content in the mortar increased rapidly with the increase in mussel shell content, and when the replacement rate of the mussel shell reached 75%, the air content was about six times higher than that of the reference mortar. This phenomenon should be attributed to the irregular and flaky shell particles. The shape of the crushed seashell affected the amount of entrapped air in the mortar, the more asymmetrical the shape, the greater amount of air in the mortar [29]. Additionally, one of the main components of mussel shells is chitin, an organic protein that was an air-producing polysaccharide [29]. Cuadrado-Rica et al. [41] also believed that changes in air content were caused by seashell shape and organic matter content. Statistics showed that there was a clear relationship between the organic matter content of the seashell and the air content of the mortar (Figure 8).

On the other hand, Edalat-Behbahani et al. [75] used an ASTM C231 [92] test method to evaluate air-entrapped air through concrete and found that replacing fine aggregate with shells had little effect on concrete air content. Yang et al. [49] also did not observe significant changes in air content when oyster shells replaced 20% of the fine aggregates. In the study by Varhen et al. [58], although up to 60% of the fine aggregates was replaced with scallop shells, there was still no noticeable change in air content. This may be due to the reduced organic content in the scallops when they were processed.

## 6. Mechanical Properties of Concrete and Mortar Containing Seashells

Mechanical properties are one of the most important properties of concrete and mortar, which determine whether it could be applied to structures. Most of the current studies are based on static experiments to obtain the mechanical properties of seashell concrete and mortar. There is a lack of research about the properties under cyclic loading. Therefore, this section would focus on the effect of seashell on the compressive strength, splitting the tensile strength, flexural strength, and static modulus of elasticity of concrete and mortar.

### 6.1. Compressive Strength

**Figure 9:**
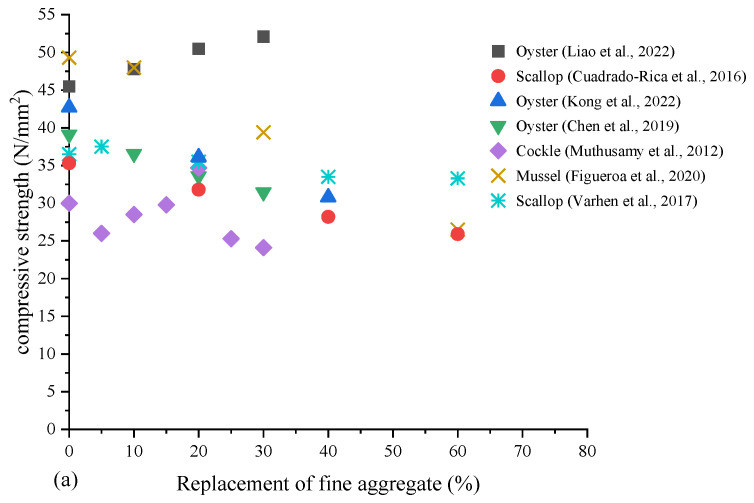

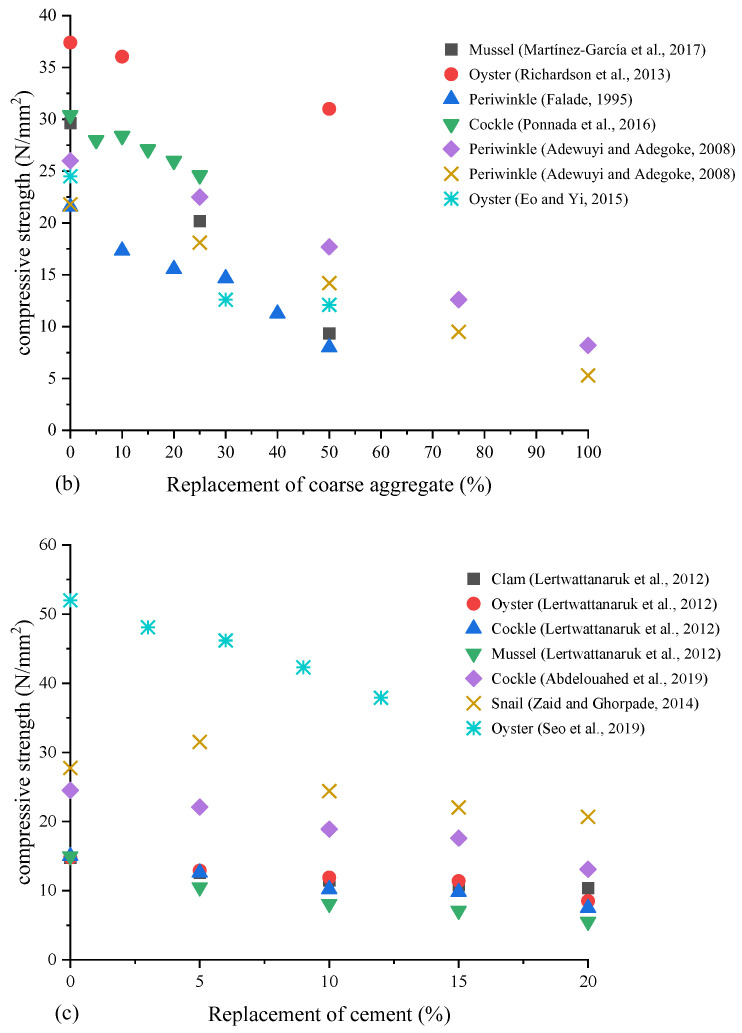
Twenty-eight-day compressive strength of cementitious materials containing waste seashells: (**a**) fine aggregate replacement; (**b**) coarse aggregate replacement; (**c**) cement replacement [20,23,25,33,40,41,42,53,56,58,62,71,76,83,84,93,94].

Compressive strength is the most important basic property of mortar and concrete and has been extensively studied in related research. The researchers considered different proportions of seashell coarse aggregate, fine aggregate and cement, and different curing periods and test conditions to achieve a comparison of strength grades. Based on the results of the study, Figure 9 shows the variation of the 28-day compressive strength of concrete and mortar containing seashell aggregate or cement. In general, the compressive strength was reduced when shells were used as aggregate substitutes.

Figueroa et al. [56] reported that the compressive strength of concrete was reduced by up to 46.2% when the fine aggregate replacement with crushed mussel shells was increased up to 60%. When cockle shells were used at up to 25% coarse aggregate replacement, the compressive strength of plain concrete was reduced by 19%, as reported by Ponnada et al. [93]. Some researchers found that the replacement of fine aggregates with seashells resulted in a reduction in the compressive strength of concrete, but the reduction was less than that caused by the replacement of coarse aggregates with seashells [43,87,95]. However, many researchers put forward the opposite view [20,71].

**Figure 10:**
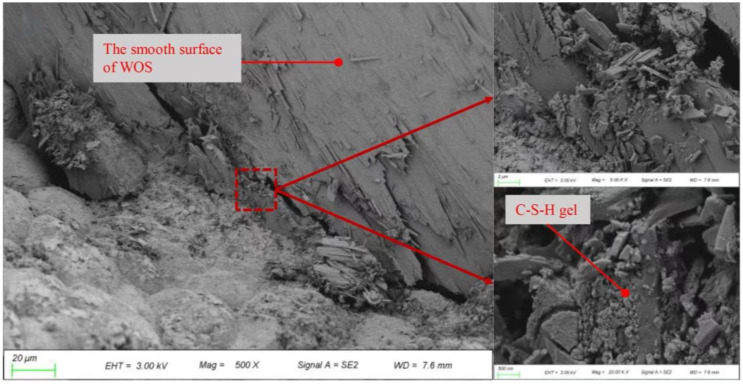
SEM of concrete: C-S-H gel grew on the smooth surface of oyster shells [42].

Martínez-García et al. [29] found that the compressive strength of the specimens decreased significantly with increasing mussel shell content. This was mainly due to the large number of macropores introduced by the irregular and flaky particle shape of mussel sand and its organic matter content. In addition, the smooth mussel shell surface developed a weak interfacial transition zone (ITZ) with the air lime matrix. In other research, Martínez-García et al. [30] pointed out that the presence of chitin in the mussel composition could damage the ITZ, thereby reducing the bond between the binder and the mussel aggregate, which could lead to a reduction in mechanical strength. As shown in Figure 10, in the bonding region, the smooth surface of the seashell hindered the close adhesion of the C-S-H gel, leading to the formation of a fragile interface around the seashell and a decreased compressive strength. Many researchers offered alternative explanations for the phenomenon that the addition of seashell aggregates reduced the compressive strength of concrete. Oh et al. [34] concluded that unwashed seashell aggregate surfaces may contain foreign matter, including organic and salt-containing substances, resulting in reduced compressive strength. Nguyen et al. [43] found that crushed shells were flat and when they were mixed together, they acted like a “wall” that prevented the particles from aligning uniformly, thereby reducing their mechanical properties. Moreover, the hydration process of concrete was disrupted by impurities and organics in the shell, resulting in bond defects that created structural defects in the cement paste [22].

In contrast, Edalat-Behbahani et al. [75] observed that the 28-day compressive strength increased by 3.8% when 100% crushed shells were used to replace the fine aggregate in the concrete. This enhancement can be attributed to the shape (angular or irregular) of seashell sands, which promoted the aggregate interlock mechanism within the concrete. Varhen et al. [58], studying the use of crushed Peruvian scallop shells as fine aggregate in concrete, suggested a maximum of 40% replacement without compromising the mechanical properties of the concrete. Moreover, it was noted that the replacement level may change due to the size and species of the particulate. Figueroa et al. [56] also suggested that in order to maintain the compressive performance of concrete, the replacement rate of fine aggregate by shell particulate should not exceed 40%.

In the investigation by Liu et al. [47], different methods of treating the crushed waste oyster shells (WOSs) noticeably affected the compressive strength of the mortar. The mortar with SS-treated (sodium silicate) crushed WOS aggregates performed better than that with the PVA-treated (polyvinyl alcohol) aggregates. This was attributed to the participation of SS in the hydration reaction as an active additive. SS contained dissolved and partially polymerized silicon that readily reacted and bound to the reaction product [96]. The reaction between SS and CH in the hydration cement slurry formed a C-S-H gel that filled the pores [97]. Liao et al. [40] found that the addition of metakaolin (MK) could increase the strength, compared to the mortar without seashell aggregate. This was due to the filling effect and the pozzolanic reaction of MK. In addition, the CaCO_3_ on the oyster shell reacted with MK to form carboaluminate, which could improve the mechanical properties. Wang et al. [28] added a large amount of ash to the oyster shell mortar to counteract the effect of seashell aggregates. However, Chen et al. [32] found that the addition of supplementary cementitious materials such as fly ash and ground granulated blast-furnace slag resulted in a decrease in the compressive strength. Luo et al. [44] added steel fibers to oyster shell concrete and found that the compressive strength of the concrete did not decrease even when all the aggregates were replaced by oyster shells. The best results were obtained at 5% concrete volume of steel fibers additive.

When seashells were used as a substitute for cement, Lertwattanaruk et al. [25] demonstrated that regardless of the type of shell, the linear strength loss increased with increasing seashell content. In the investigation by Abdelouahed et al. [83] and Lertwattanaruk et al. [25], when cement replacement with cockle shells was increased to 20%, the compressive strength was found to decrease by 50% and 46.9%, respectively. Zhong et al. [54] observed that the filler effect can improve particle packing and refined pores, so the compressive strength of mortar increased when 5% oyster shell powder was used to replace cement. However, further increasing the replacement level up to 20% resulted in a decrease in compressive strength due to the decrease in cement content [54]. This was further supported by many researchers. Abdelouahed et al. [83] found that at an early stage (2 days), the mortar with 5% cockle shell exhibited the best compressive strength. Therefore, the percentage of cockle shells must be limited to 5% or less. Similarly, Olivia et al. [66] found that the addition of 4% ground cockle shells to the mortar improved the strength development of the mortar.

In conclusion, the use of shells as a partial replacement for natural aggregates or cement has a significant influence on the compressive strength of concrete and mortar. The higher the substitution level, the lower the compressive strength of concrete and mortar. Most of the studies recommended an optimum replacement level of 20% and 5% of aggregates and cement, respectively, to achieve a strength level that meets specification requirements.

### 6.2. Splitting Tensile Strength and Flexural Strength

**Figure 11:**
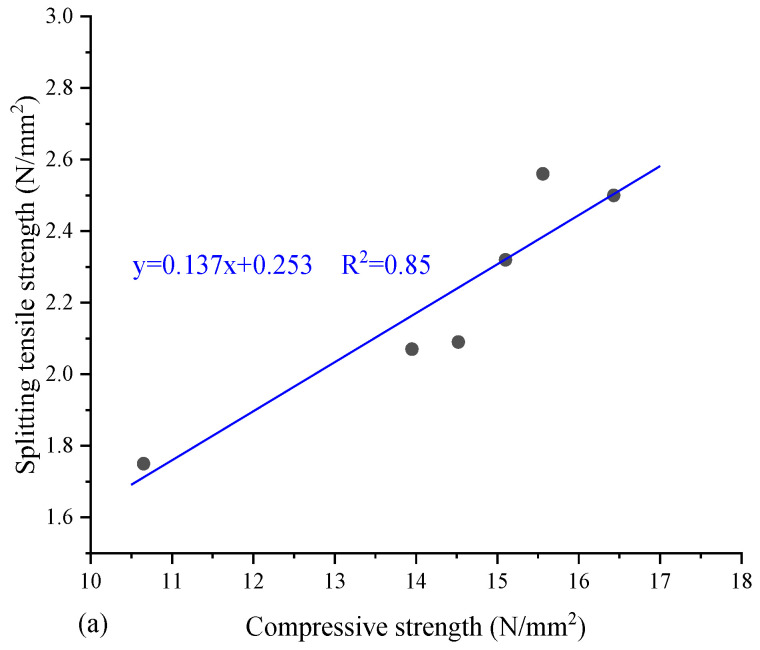

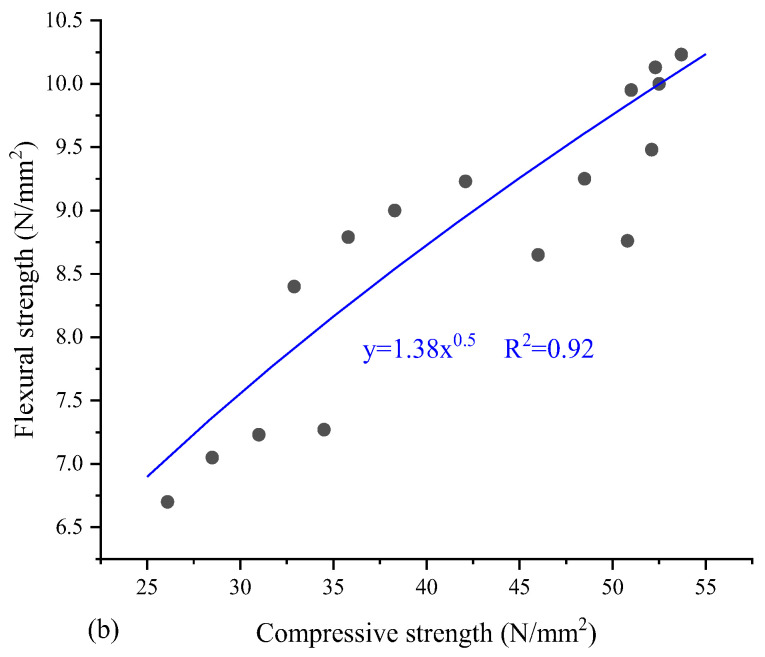
(**a**) Relationship between splitting tensile strength and compressive strength [43]; (**b**) relationship between flexural strength and compressive strength [40].

The splitting tensile strength and flexural strength of concrete or mortar are generally related to their compressive strength. Figure 11 shows that there is a certain relationship between splitting tensile strength, flexural strength and compressive strength, and researchers usually predict and evaluate other mechanical properties based on compressive strength. 

Sangeetha et al. [78] found that the tensile strength of concrete increased by 9.25% when 5% seashell powder and 10% seashell aggregates were added to the concrete. However, as the proportion of seashell powder and aggregate-replacing cement and coarse aggregate increased, the tensile strength decreased. It was suggested that a higher replacement rate would interfere with the bond between the cement paste and the aggregate. Zhong et al. [54] found that similar to the change in tensile strength, when 5% cement was replaced with oyster shell powder, the mortar reached the maximum flexural strength, and when the replacement level was increased to 20%, the flexural strength reduced by 10%. Ibrahim et al. [36] deduced the relationship between the amount of seashell powder added and the splitting tensile and flexural strength based on experimental data, and found that the seashell powder concentration that made the splitting tensile strength and flexural strength of concrete highest were 2.21% and 6.53%, respectively. Othman et al. [74] found that adding 5% cockle ash to concrete significantly improved the 90-day tensile strength, which may be due to the improved bonding at the cement paste and aggregate interface. The addition of ground shells could provide adequate bonding between the binder matrix and the aggregate at the weaker interfacial transition zone (ITZ) stage [59]. Umoh et Ujene [61] replaced cement with 30% periwinkle shells, and added NaNO_3_ as an admixture. It was observed from the experimental results that the maximum tensile strength can be obtained with 2% NaNO_3_. This showed that adding 2% NaNO_3_ to the mixture could improve the splitting tensile strength of concrete with a periwinkle shell content up to 30%.

In conclusion, the effect and mechanism of seashell addition on splitting the tensile strength and flexural strength of concrete and mortar are similar to the compressive strength. The reduction in splitting tensile and flexural strength is usually less than the compressive strength. The substitution level of seashells for aggregates and cement should still be controlled below 20% and 5%, respectively.

### 6.3. Elastic Modulus

The elastic modulus of concrete is a measure of its resistance to elastic deformation. The E-value of concrete is affected by the elastic modulus of its constituent aggregates and their volume in the concrete. Most researchers reported a reduction in the E-value of concrete and mortar when shells partially replaced aggregates or cement in concrete or mortar.

**Figure 12:**
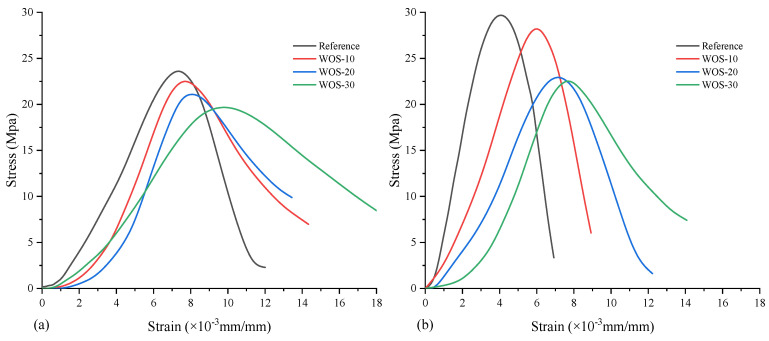
Stress–strain curves of mortars. (**a**) Curing day 28. (**b**) Curing day 90. Reference, mortar containing only river sand; WOS-10, -20, and -30, mortar samples containing 10%, 20%, and 30% crushed WOSs, respectively [33].

Yang et al. [49,70] found that when 20% of the aggregates in the concrete was replaced by crushed shells, the elastic modulus decreased by 10%. Liao et al. [87] found that when the shells replaced 20% of the aggregate, the finer the shells were ground, the greater the elastic modulus. Similar results were observed by Martínez-García et al. [20], in both structural and non-structural concrete, with coarse aggregate replacement resulting in a more severe reduction in the elastic modulus of concrete. Ahsan et al. [77] found that the loss of the elastic modulus of concrete after adding seashell aggregates was not large, mainly because the pore pressure generated in the matrix was reduced due to the decomposition of shells, which was effective in maintaining the elastic modulus. Chen et al. [33] revealed more detailed mechanical characteristics of seashell mortar by drawing the stress–strain curve based on the experimental results (Figure 12). From Figure 10, it can be found that the oyster shell as an aggregate substitute leads to a decrease in the elastic modulus of the mortar. The addition of crushed oyster shells reduces the peak stress, which is also in line with the general variation law of the compressive strength of seashell concrete and mortar in Section 5.1. Moreover, the descending stage of stress–strain curves at both curing points is flattened, indicating that the addition of crushed oyster shells reduces its brittleness and makes it more easily deformed.

## 7. Durability Properties of Concrete and Mortar Containing Seashells

### 7.1. Shrinkage and Weight Loss

Based on regression analysis, Yang et al. [70] reported that the shrinkage increased by 7% and 28% at fine aggregate replacement of 10% and 20%, respectively. Martínez-García et al. [30] found that the shrinkage of mussel shell mortars was high and increased with the percentage of added mussel shells. However, the mass loss of the mussel shell mortars was reduced, which may be due to the hindered water migration due to the particle shape of the mussel shells. According to Liao et al. [87], mortars prepared with finer crushed oyster shells had the greatest shrinkage at any curing age. This was because the finer the particles, the tighter the structure, and the larger the capillary pores in the mortar, which contributed to greater shrinkage. In addition, after curing for 90 days, the shrinkage of the mortar exceeded 0.075%, which was higher than the value required by the Standards Australia [98] so it was recommended to add more water reducer to the mortar to release the water trapped in the cement clusters. In another study by Liao et al. [38], the effect of different calcination temperatures on oyster shell mortar was investigated. The results showed that the shrinkage of oyster shell mortar was increased regardless of the calcination temperature and increased with increasing calcination temperature. This can be explained by the porosity and nature of hydrated products. At high calcination temperatures, more gel was formed, reducing the pore volume and generating more capillary pressure. Consequently, the greater water evaporation caused a greater shrinkage of mortar. Ruslan et al. [45] investigated the mass loss of seashell mortar when exposed to different temperatures. The results showed that the mass loss increased with increasing exposure temperature and seashell content. This was due to the fact that when exposed to higher temperatures, the breakdown of seashells caused porosity and diminished the strength of the mortar. Lertwattanaruk et al. [25] believed that this increase in shrinkage was due to the higher pore volume and weaker ITZ led to an increase in shrinkage age. It was also suggested that the organic matter content and the presence of chitin protein in the mussel composition led to increased shrinkage [99,100]. When the oyster shell aggregates contained the equivalent amount of fly ash and was used as the fine aggregate replacement of the mortar, the shrinkage of the mortar decreased [28]. According to the researchers, the rate of seashells as a substitute for aggregates should not exceed 20%; otherwise, the later age shrinkage will exceed the standard, whereas when seashells are used as a substitute for cement, the later age shrinkage will not increase as long as the fineness of the seashells is higher than that of the cement [25].

### 7.2. Porosity and Absorption

Based on the porosity test at 120 days, Olivia et al. [66] found that the inclusion of cockle powder decreased the porosity of the mortar by 19.3%. Through the MIP test, Liao et al. [40] justified that when the fine aggregate replacement rate of oyster shells reached 30%, the porosity of the mortar decreased by 21.3%, and the median pore diameter and porosity of the mortar decreased. Othman et al. [74] found that the porosity of concrete was reduced by 15% when cement was replaced with cockle shell powder. Artismo et al. [35] concluded that the increase in seashell content increased the virtual packing density of the granular dry mix, thus reducing porosity and absorption. This phenomenon can be attributed to the lamellar shape of mussel shell and its horizontal orientation. On the other hand, when 8%, 16%, and 33% of the cement was replaced by oyster shell powder and marine sediment, Ez-zaki et al. [55] found that the apparent porosity of all mortars increased, with a maximum increase of 11.3%. Kong et al. [42] believed that when the replacement rate of oyster shells increased from 0% to 40%, the porosity did not change much, because the oyster shells can absorb a lot of water, resulting in a lower actual water–cement ratio and more viscous paste. Viscous paste can enhance the aggregate thickness of the wrapping paste.

Chen et al. [33] found that the water absorption of mortar increased with the increase in seashell aggregate replacement level. When the oyster shell aggregate content was increased up to 30%, the water absorption of mortar was 2.4 times that of the control mortar. In another research, Chen et al. [32] reported that the addition of supplementary cementitious materials such as fly ash (FA) and ground granulated blast-furnace slag (GGBS) could significantly increase the water absorption of oyster shell mortar. Abdelouahed et al. [83] found that the water absorption decreased when the replacement rate of cockle shell in cement reached 20%, which was due to the reduction of capillary voids volume caused by the high compaction of cockle seashell mortar. However, Liu et al. [47] observed that the porosity and water absorption of crushed oyster shell aggregate mortar treated with polyvinyl alcohol (PVA) or sodium silicate (SS) were reduced compared with the control mortar.

Overall, researchers are not unanimous in their opinions on the effect of seashells on the porosity and absorption of concrete or mortar, the main reason for this discrepancy being the different particle sizes of the crushed and ground seashells. In general, it is recommended that seashells can improve durability of concrete and mortar as a replacement for aggregates and cement at rates of 10–20% and 4–10%, respectively.

### 7.3. Water Permeability

**Figure 13:**
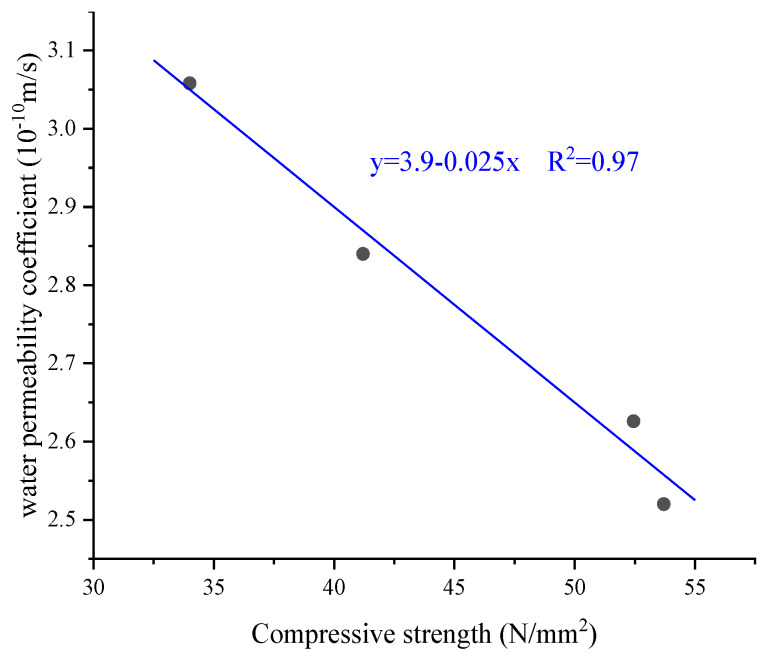
Relationship between compressive strength and water permeability coefficient [40].

Regarding the permeability coefficient of concrete, Cuadrado-Rica et al. [41] did not observe a relationship with the addition of shells as aggregate replacement. Chen et al. [33] reported that the permeability coefficient of mortar decreased rapidly with curing time and increased with the replacement level of natural fine aggregate by oyster shells. When the replacement rate reached 30%, the water permeability of mortar increased by 92.36% and 34.43% after curing for 28 days and 90 days, respectively. Liao et al. [46] investigated the effect of particle size of crushed seashells on the durability of mortar, and the results showed that the smaller the particle size of seashells, the greater the water permeability of mortar. When shells replaced up to 60% of the natural aggregate, Nguyen et al. [22] observed an increase in the water permeability of pervious concrete. Khankhaje et al. [91] and Nguyen et al. [43] also came to the same conclusion. Interestingly, Liao et al. [40] observed that the water permeability of mortar decreased with the replacement percentage of oyster shells, and it should be noted that 10% metakaolin was applied to the mortar. This was the outcome of the effect of seashell size, since the particle diameter of crushed oyster shells was smaller than that of cement, the initial pores of the mortar can be well filled and the permeability coefficient was reduced accordingly. In addition, Liao et al. [40] also demonstrated a linear relationship between water permeability and compressive strength (Figure 13). Similarly, Richardson and Fuller [71] and Martínez-García et al. [20] observed a decrease in water permeability in concrete when oyster and mussel shells were used as aggregate substitutions, respectively. According to most studies, it has been found that the strength of concrete or mortar decreases with increasing permeability; therefore, seashells cannot be substituted for more than 20% and 5% as aggregates and cement. Permeability can also be improved by adding SCMs and by reducing the particle size of the seashells after being crushed and ground.

### 7.4. Chemical Attack

Liu et al. [47] found that the chloride migration coefficient decreased with curing age, and mortars containing treated crushed oyster shell aggregates performed better in terms of chloride penetration resistance at 28 or 90 days. By testing the penetration depth of chloride ions, Abdelouahed et al. [83] observed that the penetration depth of chloride ions increased with age. On the 56th and 90th days, the mortar containing 10% cockle shells presented the best penetration resistance. On the other hand, compared with the control group, Liao et al. [40] observed a 5–10% decrease in the chloride diffusion coefficient of mortars containing oyster shells at 90 days. Ions in the pores of the cement paste, such as Ca^2+^ and Al^3+^, could react with chloride ions to form Friedel salts, which improved the microstructure of the mortar and thus reduced chloride ion diffusion. In addition, by comparing the experimental results of Chen et al. [33], it was shown that the addition of metakaolin could enhance the chloride ion diffusion resistance, protect the concrete structure containing oyster shells, and improve the resistance to chloride corrosion of marine structures. Kong et al. [42] reported that the sulfate attack resistance of concrete decreased when the oyster shell content increased from 0 to 40%. In general, the addition of seashells reduces the chemical attack resistance of concrete and mortar, but this effect can be reduced to the standard range of values as the curing time increases. In order to ensure the resistance of seashell concrete or mortar to chloride and sulphate attack, it is advisable to mix seashells as aggregates in the range of 10–20%, supplemented by special seashell treatments and the addition of SCMs.

### 7.5. Freeze–Thaw Resistance

Nguyen et al. [22] reported a 73%, 40%, and 41% reduction in the number of freeze–thaw cycles for crepidula, scallop, and queen scallop concrete, respectively, compared with control pervious concrete. The high levels of chlorides and organic matter in these shells were the most likely reasons for this finding. Yang et al. [49] believed that oyster shells as a substitute for fine aggregates in concrete could improve the freeze–thaw resistance of concrete because the oyster shell fine particles filled the trapped voids dispersed in the concrete. Li et al. [101] found that the strength loss and mass loss of the specimens under freeze–thaw cycles were minimal when the oyster shell addition was 10%. In general, the effect of oyster shell addition on freeze–thaw resistance was in relation to strength, porosity, and pore size distribution. Researchers have not reached a uniform conclusion regarding the effect of seashells on freeze–thaw resistance. In general, the effect of seashell addition on freeze–thaw resistance is related to strength, porosity, pore size distribution, particle size of seashells, and organic matter content, so it is still recommended that the addition of seashells as aggregates and cement should not exceed 20% and 5%, respectively.

## 8. Assessment of Sustainability and Economy

Reducing the environmental impact during the working process and maintenance, and improving durability at optimum engineering performance can contribute to structural sustainability. Recycling of waste seashells as aggregate or cement in concrete and mortar production offers many benefits for sustainable development. The sustainability of the seashell concrete and mortar mixes was assessed by equivalent CO_2_ emissions. In sustainability studies on seashell concrete, the CO_2_ emissions of cement, seashells, river sand, and other added materials were generally considered. In the CO_2_ emissions calculations, the CO_2_ produced by the seashell transport and the usage of seashells during the experiment was not taken into account. According to the study by Liao et al. [40], the CO_2_ equivalent emissions for producing one cubic meter of oyster shell mortar are shown in Table 8. In the Table 8, the “control” represents mortar prepared with river sand only; WOS-10, -20, and -30, represent mortar prepared with 10%, 20%, and 30% crushed waste oyster shells, respectively. The statistic shows that compared with the control mortar, when the oyster shells partially replace the aggregates in the mortar, the total CO_2_ emissions can be reduced to a certain extent, and with the increase in oyster shell dosage, the CO_2_ emission gradually decreases. Therefore, by recycling waste seashells into concrete or mortar as a renewable material, not only the area occupied by waste seashells and the pollution to the environment can be reduced, but also the cleaner production of environmentally friendly sustainable concrete can be realized.

The substitution of natural material by waste seashell not only improves sustainability, but also brings considerable economic benefits. In concrete and mortar production, the cost of river sand mainly comes from the mining process, while the cost of seashells mainly comes from the collection and production process. The unit prices of raw materials for preparing mortar obtained in the southeastern coastal areas of China are shown in Table 9. According to the data in Table 9 and Table 10, crushed oyster shells are 38.5% cheaper than river sand, and oyster shells added with 30% are about 5.75% cheaper than the reference mortar [33]. In another study by Chen et al. [32], the addition of waste shells can save up to 13.72% of the cost. Liao et al. [40] found that replacing natural aggregate and cement with 30% shells and 10% metakaolin, respectively, can reduce the cost by about 2.2% per kilogram of mortar. Therefore, it is not difficult to see that although the cost of materials varies around the world, the use of waste shells as an alternative to aggregate or cement can improve the cost efficiency of concrete and mortar as a whole.

In fact, there are also some disadvantages to exploit seashells. For example, when seashells are used as a substitute for cement, an additional calcination process is required, which could generate additional CO_2_ emissions and costs. However, taking into account the ecological benefits of recycling waste seashells, as well as the overall reduction in CO_2_ emissions and costs, we believe that the use and exploitation of waste seashells as aggregate or cement substitutes is both sustainably and economically beneficial.

## 9. Conclusions

- The main component of seashells is calcium carbonate, so it can be considered an inert material and added to concrete or mortar in aggregate or powder form. The specific gravity of seashell aggregate is usually lower than that of natural aggregates while the water absorption is higher. Since seashells come mainly from waste, which usually contain many impurities, proper treatment should be carried out before reused. In general, the treatment of waste seashells involves cleaning, drying, and crushing to remove significant amounts of chlorides, sulfates, and organic matter. Some researchers also perform additional calcination treatment.
- Studies have shown that in most cases, the incorporation of seashell aggregate will adversely affect the workability of concrete and mortar, which is mainly due to the high porosity and water absorption, angular shape, and rough surface of seashells. Similarly, the addition of seashells also prolongs the setting time and reduces the density of concrete and mortar. In terms of the mechanical properties of concrete and mortar, the use of shells as a substitute for aggregate reduces the mechanical properties to a certain extent. Although the strength of seashell concrete is satisfied, the lower limit for structural purposes. But given the difficulty of meeting the standards for its workability and chloride ion content, there still exists uncertainty about the use for structural purposes from the point of engineering safety. Consequently, shell concrete can still only be used for non-structural purposes. With the addition of seashell aggregates, the resistance to a chemical attack generally decreases and the shrinkage properties increase. However, there is still a lack of agreement on certain aspects of durability such as water permeability, freeze–thaw resistance, and porosity which depends largely on the particle size of the seashell aggregates, the particular method of treatment, the addition of other SCMs and other factors in their tests. Therefore, there is an optimum value for the amount of seashells. According to the results of most researchers, the replacement rate of seashell as aggregate should generally be limited to less than 20%.
- There is insufficient literature to summarize the effects of seashell powder as cement replacement on the various properties of concrete and mortar. In general, the addition of seashell will reduce the workability of concrete or mortar because it increases the water demand. Similarly, the setting time of the concrete or mortar increases and the density decreases slightly. Partial replacement of cement with seashell reduces the compressive strength of the concrete or mortar, but has a beneficial effect on splitting tensile strength and flexural strength at low levels of replacement. Similarly, concrete and mortar with using seashells as cement can only be used for non-structural purposes. Conversely, the addition of seashell will reduce the shrinkage of the concrete or mortar. As with seashell as aggregates, there is still a lack of consistent conclusions on other durability aspects. Therefore, the replacement rate of seashell as cement should be limited to about 5%.
- Overall, the use of seashells in concrete and mortar has good potential. Recycling waste seashells can not only reduce the environmental impact of such waste produced by shellfish, but also decrease the dependence on raw materials in the construction industry and promote its sustainable development.

## 10. Directions for Future Investigations

Although seashell concrete has been tested and proved its potential application in the construction industry, there are still several important problems that need further research. Based on the existing research results, this literature review puts forward the following suggestions for future research directions:

- Most of the current trials were carried out in the laboratory and had limited trial time, whereas long-term mechanical and durability field trials are more informative for practical engineering applications. In future studies, longer trials are needed to be conducted to evaluate durability more precisely.
- Current research lacks the properties of seashell concrete and mortar under cyclic loading. Therefore, in order to make a more comprehensive assessment of their suitability in structures, future research needs to focus on the properties under cyclic loading, such as dynamic modulus of elasticity, and fatigue resistance.
- For seashell concrete, the research on durability is very important. Although some conclusions have been available in the literature on the durability of seashell concrete, it often faces more complex environmental conditions when it is applied in practical engineering. There is a lack of research in previous studies on the fire resistance, high-temperature resistance, thermal insulation, sound absorption, carbonization, etc. of seashell concrete, so further research in these areas of durability is needed.
- The research on chemical resistance of seashell concrete is only limited to a single resistance to chloride or sulfate corrosion. However, the results of previous research in this area do not guarantee its use in practical structures, as the actual marine conditions faced in coastal areas are more complex. Thus, it is necessary to research the properties of seashell concrete under the combined erosion of chloride and sulfate.
- The current research is still at the stage of plain concrete. The interaction between seashell concrete and rebars in bond-slip and other aspects are still unclear, so current research cannot guarantee that seashell concrete can be used in reinforced concrete members to extend its further application. Therefore, future research needs to focus on the performance of reinforced concrete beam and column containing seashells to further increase the possibility of their engineering applications.
- There are few ecological and economic feasibility assessments of seashell concrete and mortars to determine the feasibility of commercial-scale implementation of the use of seashells to replace aggregates in mass concrete. Therefore, research needs to pay more attention to the eco-efficiency and cost-efficiency of seashell concrete.

## Figures and Tables

**Table 1:** The 10 most common keywords in the literature.

No.	Keyword	Amount
1	Concrete	322
2	Cement	266
3	Compressive strength	194
4	Oyster shell	185
5	Strength	162
6	Fly ash	125
7	Fine aggregate	111
8	waste	90
9	Mechanical properties	78
10	Durability	75

**Table 2:** Physical properties of seashell waste as aggregate.

Seashell Type	Literature	Size (mm)	Fineness Modulus	Specific Gravity	Water Absorption (%)
Oyster	Yang et al. [49]	<5	2.80	2.48	2.90
Oyster	Kuo et al. [50]	<4.75	2.00	2.10	7.70
Oyster	Islam et al. [51]	<2	2.27	2.29	-
Oyster	Eo and Yi [23]	<5	1.85	2.59	1.61
		25	7.68	2.67	0.40
Oyster	Chen et al. [32]	<5	3.66	-	6.84
Oyster	Chen et al. [33]	<5	3.72	-	8.87
Scallop	Cuadrado-Rica et al. [41]	<5	4.40	2.64	3.65
Mussel	Martínez-García et al. [20]	0–1	1.90	2.73	4.12
		1–4	4.64	2.65	2.56
		4–16	5.38	2.62	2.17
Cockle	Khankhaje et al. [24]	4.75–6.3	-	2.64	2.50
		6.3–9.5	-	2.09	1.80

**Table 3:** Chemical composition of seashells (%).

Seashell Type	Literature	CaCO_3_/CaO	SiO_2_	Al_2_O_3_	MgO	Fe_2_O_3_	Na_2_O	K_2_O	SO_3_	P_2_O_5_	LOI
Raw shells											
Seashell	Abinaya and Venkatesh [26]	89.56	4.04	0.42	0.65	-	0.98	-	0.72	0.20	-
River shell		95.99	1.28	0.40	0.68	-	0.98	-	072	0.20	-
Oyster	Kong et al. [42]	95.32	1.01	0.26	0.71	0.15	1.18	-	0.66	-	-
Mussel	Figueroa et al. [56]	96.9	1.30	-	-	0.50	-	0.40	0.30	-	-
Cockle	Oh et al. [34]	97.6	0.13	0.10	0.32	0.28	1.22	0.03	0.12	-	-
After calcination											
Oyster	Yang et al. [49]	51.06	2.00	0.50	0.51	0.20	0.58	0.06	0.60	0.18	44.16
Oyster	Jung et al. [57]	53.81	0.40	0.22	0.70	0.04	-	-	-	-	44.87
Scallop	Varhen et al. [58]	53.70	0.10	0.10	0.18	0.03	0.50	0.01	0.32	-	44.4
Mussel	Jung et al. [57]	53.70	0.20	0.13	0.33	0.03	-	-	-	-	45.61
Mussel	Felipe-Sese et al. [27]	87.21	0.55	0.03	0.49	0.05	0.50	0.04	-	0.09	-
Cockle	Olivia et al. [59]	51.56	1.60	0.92	1.43	-	0.08	0.06	-	-	41.84
Cockle	Olivia et al. [21]	51.91	0.38	0.65	-	0.05	-	-	-	-	-
Clam	Jung et al. [57]	53.92	0.46	0.20	0.22	0.04	-	-	-	-	45.16
Clam	Olivia et al. [21]	67.70	0.39	0.28	-	0.02	-	-	-	-	-
Periwinkle	Etuk et al. [60]	55.53	26.26	8.79	0.40	4.82	0.25	0.20	0.18	0.05	-
Periwinkle	Umoh and Ujene [61]	52.10	27.20	6.42	0.82	4.64	0.26	0.25	0.26	-	-
Snail	Zaid and Ghorpade [62]	51.09	0.60	0.51	0.69	0.56	1.20	0.12	0.19	0.21	40.54
Cardiidae	Soltanzadeh et al. [63]	52.34	3.65	1.15	0.42	0.20	0.35	0.13	0.47	-	41.25

**Table 4:** Methods of preparation of seashell concrete or mortar reported in the literature.

Seashell Type	Literature	Mixture Type	Replaced Material	w/c	Percentage (%)	Desin Mix
Oyster	Liao et al. [40]	mortar	Fine aggregate	0.45	10, 20, 30	1:2.5
Oyster	Liu et al. [47]	mortar	Fine aggregate	0.45	20	1:2.5
Mussel	Martínez-García et al. [29]	mortar	Fine aggregate	1.77 (by volume)	25, 50, 75 (by volume)	-
Mussel	Martínez-García et al. [64]	mortar	Fine aggregate	0.64	25, 50, 75	1:2.02
Cockle	Edalat-Behbahani et al. [75]	mortar	Fine aggregate	0.45 0.4	30	1:4.5 1:2.5
Cockle	Khankhaje et al. [24]	concrete	Coarse aggregate	0.32	25, 50, 70	1:0.41:3.93
Cockle	Muthusamy and Sabri [76]	concrete	Coarse aggregate	0.5	5, 10, 15, 20, 25, 30	-
Cockle	Olivia et al. [66]	mortar	Cement	0.55	4	1:2.75
Cockle	Othman et al. [74]	concrete	Cement	0.54	5, 10, 15, 25, 50	1:2.5:1.35
Periwinkle	Falade [53]	concrete	Coarse aggregate	0.55 0.6 0.8	10, 20, 30, 40, 50	1:1.5:3 1:2:4 1:3:6
Not specified	Ahsan et al. [77]	concrete	Fine aggregate	0.35	10, 20, 30	1:1.35:2.8
Not specified	Sangeetha et al. [78]	concrete	Cement Coarse aggregate	0.5	5, 10, 15 10, 20, 30	1:1.6:3.4
Not specified	Hasan et al. [67]	mortar	Cement	0.49	5, 10, 15, 20, 25, 30	1:2.54

**Table 5:** The effect of seashells on workability.

Literature	Replaced Material	Replacement Level	Degree of Impact
Liao et al. [40]	Fine aggregate	10%	−25%
		20%	−32.5%
		30%	−36.1%
Adewuyi et al. [84]	Coarse aggregate	25%	−26.7%
		50%	−46.7%
		75%	−66.7%
Hazurina et al. [68]	Cement	5%	−33.7%
		10%	−66.7%
		15%	−88.7%

**Table 6:** The effect of seashells on setting time.

Literature	Replaced Material	Replacement Level	Degree of Impact	
			Initial	Final
Wang et al. [89]	Fine aggregate	5%	+10.8%	+29.7%
		10%	+23.9%	+49.4%
		20%	+34.7%	+61.3%
		30%	+49.7%	+76.5%
Lertwattanaruk et al. [25]	Cement	5%	+1.7%	+1.1%
		10%	+6.9%	+2.2%
		15%	+8.6%	+6.7%
		20%	+10.3%	+13.3%
Hazurina et al. [68]	Cement	5%	+66.7%	+19.1%
		10%	+100.0%	+28.6%
		15%	+100.0%	+38.1%
		25%	+111.1%	+47.6%
		50%	+122.2%	+61.9%

**Table 7:** The effect of seashells on hardened density.

Literature	Replaced Material	Replacement Level	Degree of Impact
Cuadrado-Rica et al. [41]	Fine aggregate	20%	−1.67%
		40%	−6.69%
		60%	−6.95%
Chen et al. [33]	Fine aggregate	10%	−0.24%
		20%	−0.95%
		30%	−1.43%
Ez-zaki et al. [55]	Cement	8%	−0.21%
		16%	−1.23%
		33%	−2.21%

**Table 8:** Total CO_2_ released in oyster shell mortar [40]. Adapted with permission.

Mix	CO_2_ Emissions (kg CO_2_/m^3^)						Total CO_2_ Emissions (kg CO_2_/m^3^)
	Cement	Metakaolin	River Sand	Oyster Shells	SP	Water	
Control	419.70	3.65	4.56	0	1.13	0	429.04
WOSP-10	419.70	3.65	4.11	0.30	1.13	0	428.89
WOSP-20	419.70	3.65	3.66	0.60	1.13	0	428.74
WOSP-30	419.70	3.65	3.21	0.90	1.13	0	428.59

**Table 9:** Unit cost of mortar materials [33].

	Cement	River Sand	Oyster Shells	Superplasticizer	Water
Unit cost (USD/kg)	0.074	0.031	0.019	1.07	0.00055

**Table 10:** Cost efficiency of different mortar mixtures [33].

Mix	Raw Material Cost (USD/m^3^)					Total Material Cost (USD/m^3^)
	Cement	River Sand	Oyster Shells	Superplasticizer	Water	
Reference	44.83	46.60	0.00	1.95	0.15	93.53
WOS-10	44.83	41.94	2.87	1.95	0.15	91.74
WOS-20	44.83	37.28	5.74	1.95	0.15	89.95
WOS-30	44.83	32.62	8.60	1.95	0.15	88.15

Reference, mortar mixture prepared with river sand only; WOS-10, -20, and -30 mortar mixtures prepared with 10%, 20%, and 30% crushed WOSs, respectively.

## Data Availability

Data will be made available on request (due to privacy).
